# Protective role of integrin-linked kinase against oxidative stress and in maintenance of genomic integrity

**DOI:** 10.18632/oncotarget.24444

**Published:** 2018-02-07

**Authors:** Michelle Im, Lina Dagnino

**Affiliations:** ^1^ Department of Physiology and Pharmacology, The University of Western London, Ontario, Canada; ^2^ Lawson Health Research Institute, London, Ontario, Canada; ^3^ Children's Health Research Institute, London, Ontario, Canada

**Keywords:** oxidative stress, epidermis, integrin-linked kinase

## Abstract

The balance between the production of reactive oxygen species and activation of antioxidant pathways is essential to maintain a normal redox state in all tissues. Oxidative stress caused by excessive oxidant species generation can cause damage to DNA and other macromolecules, affecting cell function and viability. Here we show that integrin-linked kinase (ILK) plays a key role in eliciting a protective response to oxidative damage in epidermal cells. Inactivation of the *Ilk* gene causes elevated levels of intracellular oxidant species (IOS) and DNA damage in the absence of exogenous oxidative insults. In ILK-deficient cells, excessive IOS production can be prevented through inhibition of NADPH oxidase activity, with a concomitant reduction in DNA damage. Additionally, ILK is necessary for DNA repair processes following UVB-induced damage, as ILK-deficient cells show a significantly impaired ability to remove cyclobutane pyrimidine dimers following irradiation. Thus, ILK is essential to maintain cellular redox balance and, in its absence, epidermal cells become more susceptible to oxidative damage through mechanisms that involve IOS production by NADPH oxidase activity.

## INTRODUCTION

Given its location at the interface between the body and the environment, the epidermis is constantly in direct contact with oxidants, such as atmospheric oxygen, air pollutants including ozone and airborne metal-containing particles, and UV radiation. The epidermis is, in fact, a tissue exposed to high levels of oxidative damage in large part due to the generation of reactive oxygen species (ROS) [1, 2]. Although ROS play important physiological roles in signalling and normal activation of immune responses, excessive ROS production can lead to harmful oxidation of proteins, lipids and DNA [3].

Several protective mechanisms against oxidative damage exist in the epidermis. Cysteine residues in the small proline-rich family of the cornified envelope precursor proteins are readily oxidized by ROS, thereby serving as a shield in the outermost layer of this tissue [4]. In addition, within the living layers of the epidermis, various antioxidant molecules are actively synthesized, including glutathione, α-tocopherol and ascorbic acid, as well as a variety of detoxifying enzymes (reviewed in [2]). Elevated oxidative stress resulting from impaired antioxidant mechanisms or excessive ROS production can give rise to a plethora of cutaneous disorders. For example, increased ROS levels are implicated in the pathogenesis of inflammatory diseases such as psoriasis, in atopic dermatitis and development of allergic reactions in the skin, in drug-induced photosensitization and photodamage [2, 5, 6]. Photodamage from UV radiation is a major cause for cellular senescence, age-associated decreases in epidermal function and regeneration, as well as cutaneous tumour development. UV damage promotes initiation and progression of epidermal carcinomas through mechanisms that include ROS-induced metabolic, genetic and epigenetic alterations in keratinocytes. In particular, activation and upregulation of the ROS-producing enzyme NOX1 is a key step in the formation of epidermal squamous cell carcinomas in response to UV radiation [7].

Recent studies have demonstrated that appropriate regulation of redox balance is key to the maintenance of epidermal integrity. For example, in the human genetic disease pachyonychia congenita, and in mouse models of this disorder, elevated oxidative stress occurs through mechanisms that involve attenuated nuclear factor erythroid-derived related factor-2 (NRF2) transcriptional activity, with a consequent reduction in the expression of enzymes involved in glutathione synthesis [8]. Similarly, in Kindler syndrome, a familial blistering disease, inactivating mutations in the *FERMT1* gene, which encodes Kindlin-1, result in loss of epidermal attachment and integrity, as well as susceptibility to cutaneous squamous cell carcinomas [9]. Kindlin-1-deficient keratinocytes exhibit higher cellular ROS levels and increased UV-induced DNA damage [10].

Integrin-linked kinase is a ubiquitous scaffold protein that also plays key roles in the maintenance of epidermal integrity and barrier function [11–14]. In keratinocytes, ILK modulates microtubule dynamics, development of front-rear polarity, endosomal trafficking, and forward motility [15–19]. ILK associates with the β1 integrin adhesome, and epidermis-restricted inactivation of the *Ilk* gene results in keratinocyte detachment from the underlying basement membrane through mechanisms that include abnormalities in cellular integrin distribution and signalling [20]. Perturbations in integrin-mediated functions can lead to loss of keratinocyte extracellular matrix deposition and defects in matrix attachment [20]. Integrins and their associated proteins play complex roles in the modulation of oxidant species production. For example, integrin engagement cooperates with growth factor signalling in epithelial cells to activate survival pathways through mechanisms that include ROS generation [21]. Conversely, loss of integrin signals can lead to pathologic stimulation of ROS production, metabolic alterations and anoikis [10, 22]. A key unresolved issue is whether ILK is also involved in controlling cellular redox balance and responses to oxidative stress. We now show that ILK is necessary to maintain normal intracellular oxidant species levels (IOS), thereby protecting cells from oxidative damage. We also demonstrate an essential role for ILK in the repair of DNA lesions following UV irradiation.

## RESULTS

### ILK deficiency increases keratinocyte susceptibility to apoptosis under serum deprivation conditions

**Figure 1:**
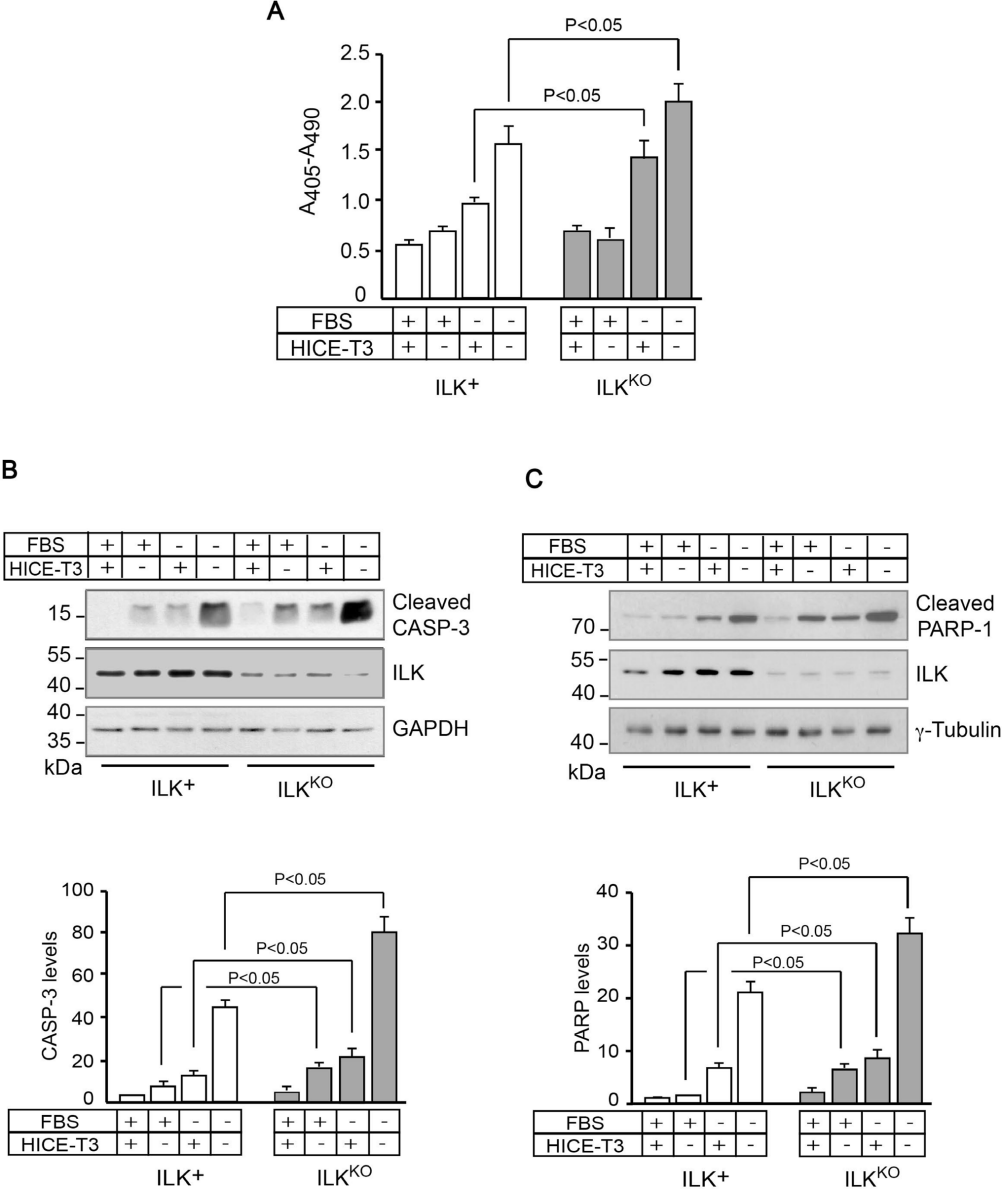

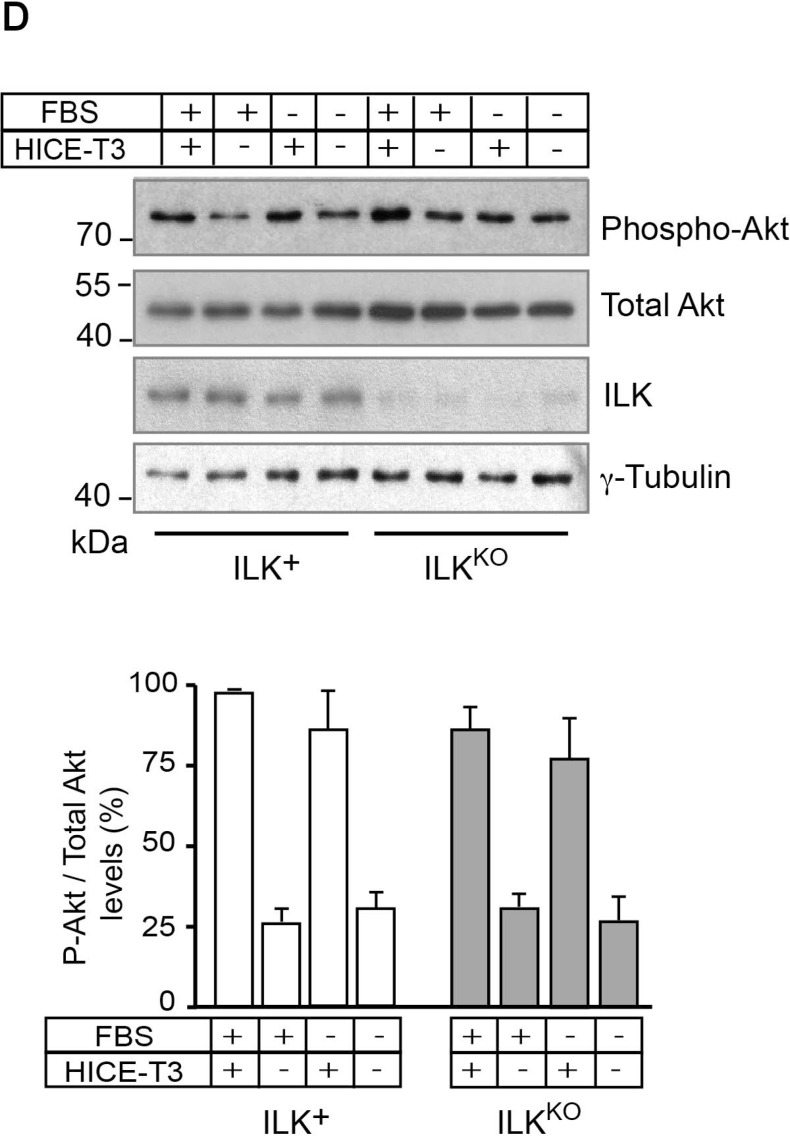
Increased sensitivity of ILK^KO^ keratinocytes to growth factor deprivation ILK^+^ and ILK^KO^ keratinocytes were cultured in the presence of the indicated additives for 24 h. (**A**) Cytoplasmic mono- and oligonucleosomes in keratinocyte lysates were quantified using a colorimetric enzyme-linked immunosorbent assay method. The results are expressed as mean absorbance at 405 nm (reference wavelength 490 nm) ± SEM (*n* = 7). Statistical significance was calculated using ANOVA. (**B**–**D**) Cell lysates were analyzed by immunoblot using antibodies against the indicated proteins, and GAPDH or γ-tubulin served as loading controls. The histograms below each representative immunoblot show normalized densitometric quantification of each protein (mean ± SD; *n* = 3), and are expressed relative to the abundance in ILK^+^ cells cultured in growth medium with FBS and HICE-T3, which is set to 1.0. Statistical significance was calculated using ANOVA. CASP-3, caspase 3.

To investigate whether ILK regulates keratinocyte survival under various conditions, we measured levels of cytoplasmic oligonucleosomes, indicative of apoptosis, in cells isolated from ILK-expressing (ILK^+^) or ILK-deficient (ILK^KO^) epidermis. ILK^KO^ cells cultured under optimal conditions, consisting of growth medium supplemented with fetal bovine serum (FBS), as well as growth and survival factors (HICE-T3, containing hydrocortisone, insulin, cholera toxin, epidermal growth factor and tri-iodothyronine), exhibited similar levels of apoptosis-associated oligonucleosomes to ILK^+^ cells (Figure 1A). However, when ILK^KO^ cells were cultured in serum-deficient medium, they showed significantly higher levels of oligonucleosomes than ILK^+^ keratinocytes, irrespective of whether HICE-T3 factors were present or absent in the culture medium. This indicates a greater susceptibility of ILK-deficient keratinocytes to the pro-apoptotic effects of serum deprivation (Figure 1A). Biochemically, ILK^KO^ keratinocytes also showed increased levels of active, cleaved caspase 3, relative to normal cells, when they were cultured in the absence of serum and/or growth factors (Figure 1B), indicating that the absence of ILK results in activation of intrinsic apoptosis pathways under those stress conditions. Consistent with this notion, increased levels of an 89-kDa cleavage fragment of PARP-1, generated by caspase-mediated proteolysis, were also present in ILK^KO^ cells (Figure 1C). Significantly, the abundance of phosphorylated Akt was indistinguishable between ILK+ and ILK^KO^ cells (Figure 1D), suggesting that ILK is dispensable for Akt phosphorylation, and that the PI3-kinase-Akt survival signalling pathway does not likely contribute significantly to the observed differences in the sensitivity to apoptotic stimuli between ILK-expressing and ILK^KO^ keratinocytes.

### Decreased proliferative capacity of ILK^KO^ keratinocytes

**Figure 2:**
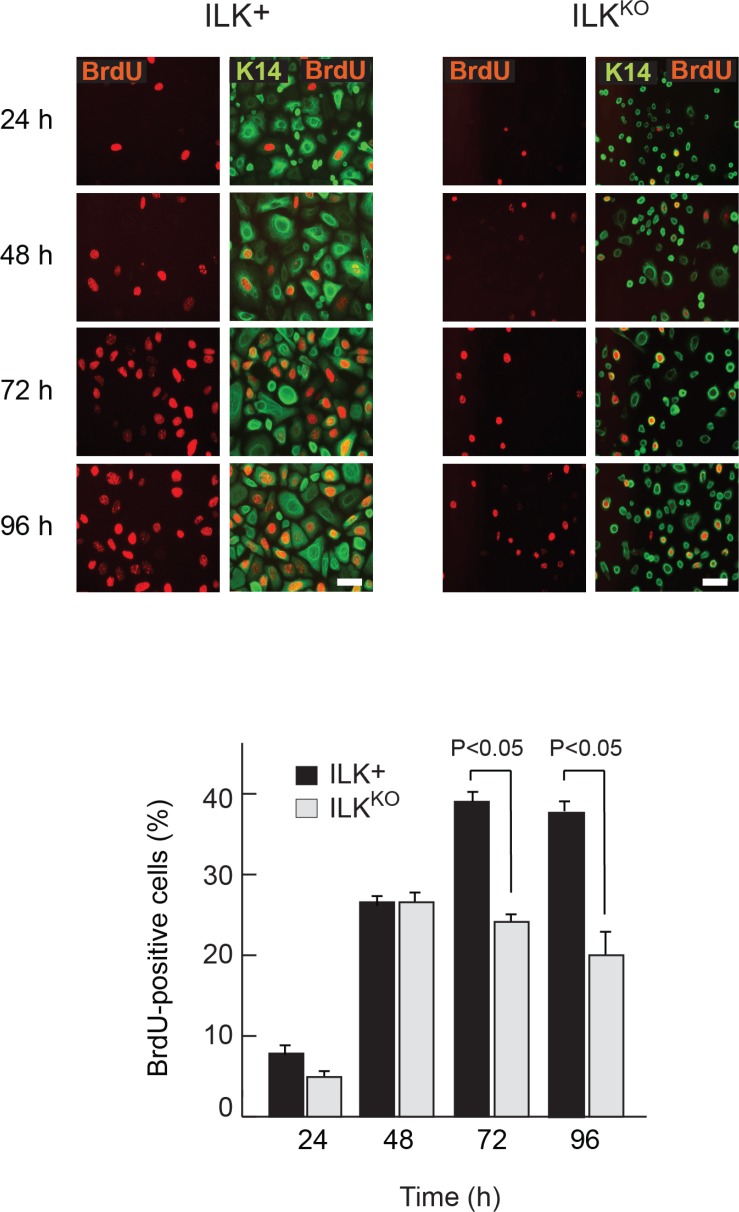
Proliferation defects in ILK^KO^ keratinocytes Representative micrographs of keratinocytes incubated in the presence of 10 μM BrdU for 2 h prior to processing for immunofluorescence microscopy using anti-BrdU and anti-keratin 14 (K14) antibodies at the indicated times following isolation and plating. Bar, 60 µm. The histograms indicate the fraction of BrdU-positive cells of each genotyope, represented as the mean + SEM (*n* = 3). Statistical significance was calculated using ANOVA.

The abnormalities in ILK^KO^ keratinocytes in response to apoptotic stimuli prompted us to investigate if these cells also showed defects in proliferative capacity in the presence of mitogens, by measuring BrdU incorporation into DNA. The percentage of BrdU-positive ILK-expressing cells steadily increased from <5% to about 40% by 72 h following isolation and culture in normal growth medium. This proportion remained steady once the cell population started to proliferate asynchronously, after 96 h in culture (Figure 2). In contrast, although the proportion of ILK^KO^ keratinocytes that showed BrdU incorporation initially increased 24 h after plating, it failed to increase any further, suggesting that normal responses to mitogenic factors present in the culture medium are impaired in these cells (Figure 2). Of note, loss of ILK results in spreading defects in cultured keratinocytes [23], and impaired cell spreading in these cells is associated with entry into quiescence and activation of differentiation programs involving Notch signaling [24].

**Figure 3:**
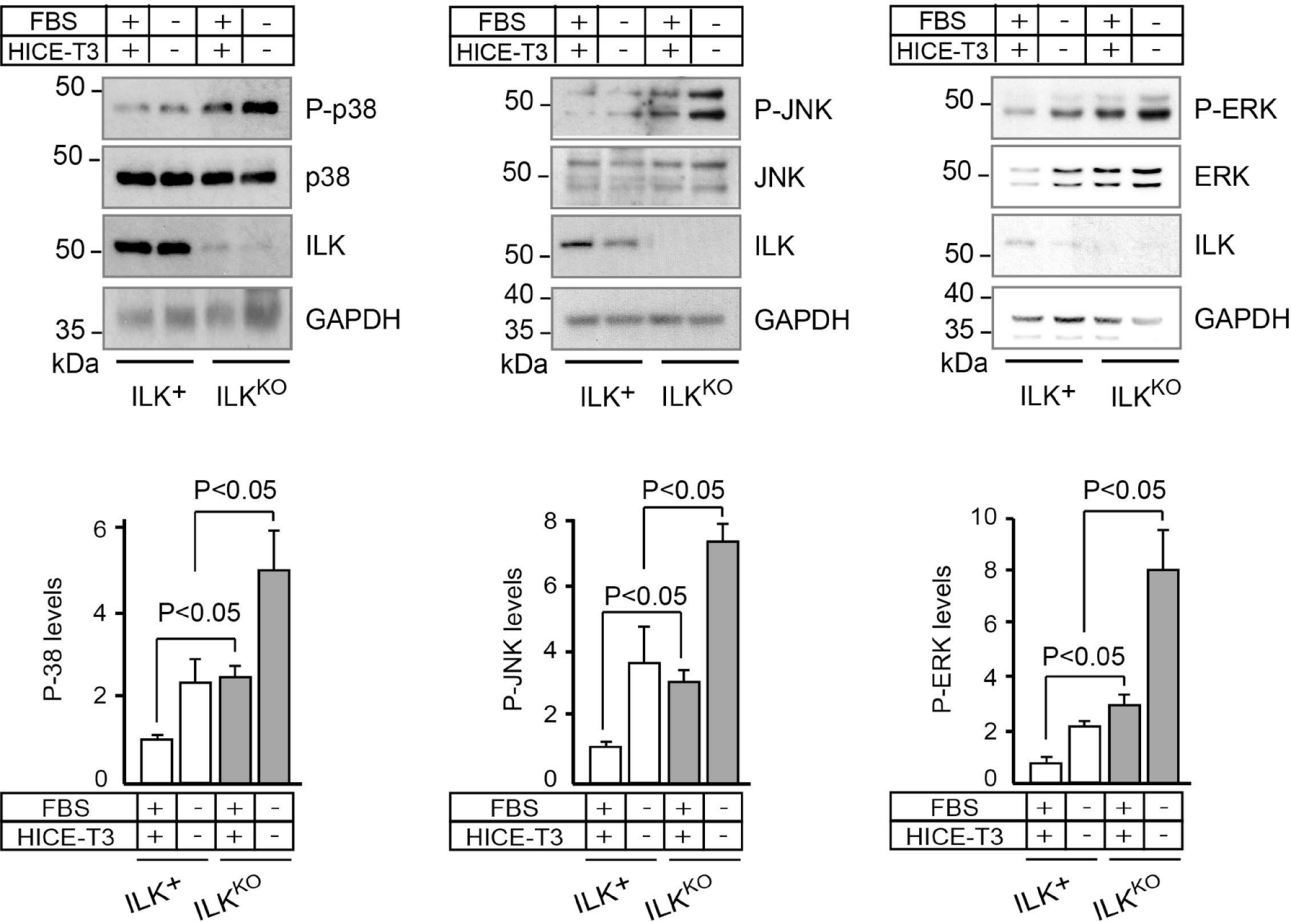
Increased levels of phosphorylated MAP kinases in ILK^KO^ keratinocytes Keratinocytes were cultured in the presence or absence of growth additives. After 24 h, cell lysates were prepared and analyzed by immunoblot using antibodies against phosphorylated (P-) or total cellular p38, JNK or ERK; GAPDH served as loading control. The histograms under each representative immunoblot show the densitometric quantification of each phosphorylated protein normalized to the corresponding levels of total protein (mean ± SD; *n* = 4). Statistical significance was calculated using ANOVA.

The mammalian mitogen-activated protein kinase (MAPK) pathway includes three main arms: p38, JNK and ERK. Multiple stimuli and stressors activate MAP kinases in keratinocytes and other cell types, including growth factors, hypoxia, UV irradiation and oxidative stress [25–27]. Thus, given the defects in responses to mitogens observed in ILK^KO^ cells, we next examined the relationship between loss of ILK and MAP kinase activation. Significantly, levels of active, phospho-p38, phospho-JNK, and phospho-ERK were 2- to 8-fold higher in ILK^KO^ cells, irrespective of whether the cells were cultured in the presence or absence of growth factors and serum (Figure 3). These observations suggest that ILK deficiency does not impair MAP kinase activation *per se*. However, the alterations in the proliferation profile of ILK-deficient keratinocytes may be influenced, at least in part, by abnormal activation and/or impaired dephosphorylation of these enzymes, in addition to stress stimuli possibly present in these cells.

### Elevated IOS levels in the absence of ILK

**Figure 4:**
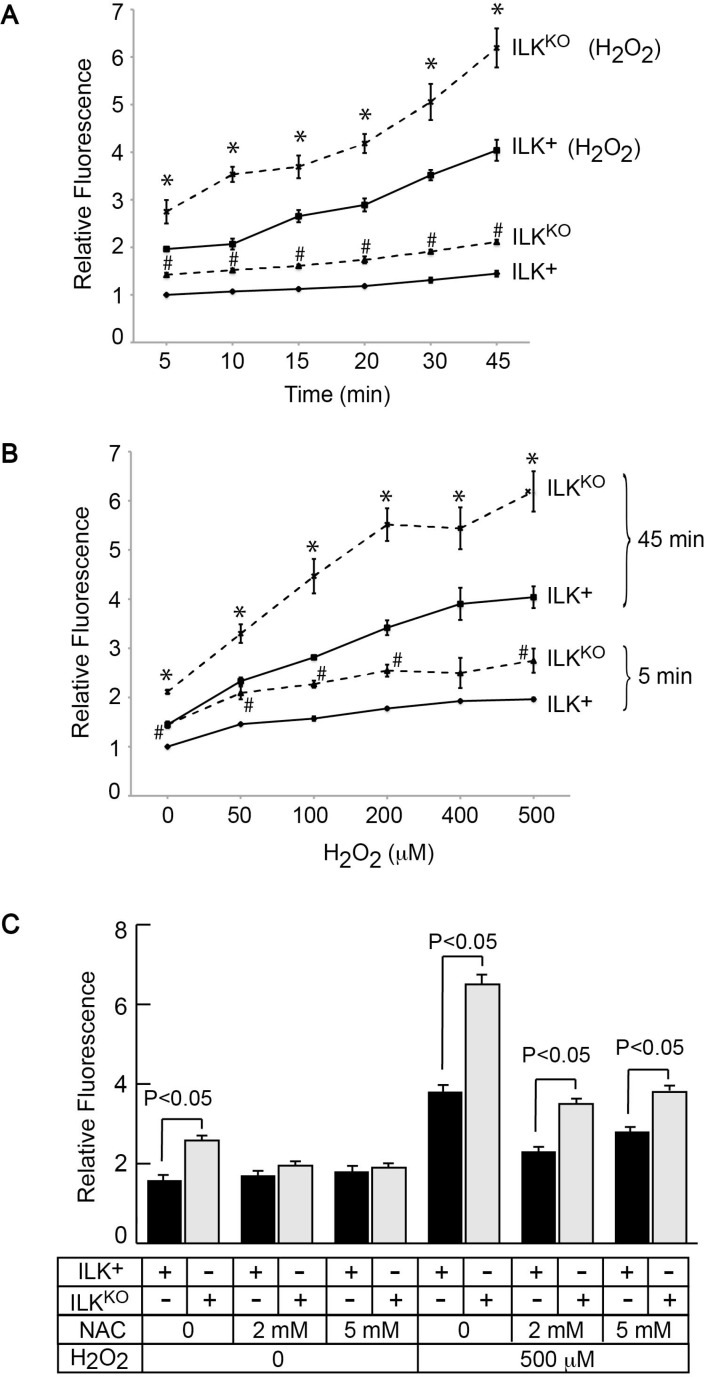
Increased intracellular oxidant species in ILK^KO^ keratinocytes Cells were loaded by culture at 37° C in the presence of 10 μM DCFDA. (**A**) After DCFDA removal, fluorescence values were determined at the indicated times in cultures incubated with or without 500 μM H_2_O_2_, and were normalized for cell number. The results are expressed as fold-change relative to the 5-min normalized fluorescence value in ILK^+^ cultures incubated without H_2_O_2_, which is set to 1. Data are means ± SEM (*n* = 3). ^#^ and ^*^represent, respectively, *P* < 0.05 relative to ILK^+^ cells in the absence or presence of H_2_O_2_ at the corresponding time (ANOVA). (**B**) After DCFDA removal, fluorescence values were determined after 5 or 45 min in cultures incubated with the indicated H_2_O_2_ concentrations, and were normalized for cell number. The results are expressed as fold-change relative to the 5-min normalized fluorescence value in ILK^+^ cultures incubated without H_2_O_2_, which is set to 1. Data are means ± SEM (*n* = 3). ^#^ and ^*^represent, respectively, *P* < 0.05 relative to ILK^+^ cells in the absence or presence of H_2_O_2_ at the corresponding time (ANOVA). (**C**) Keratinocytes were incubated with the indicated NAC concentrations for 45 min and, after NAC removal, were loaded with DCFDA and fluorescence was measured as described in (B). The results are expressed as fold-change in cultures measured after 45 min, relative to the 5-min normalized fluorescence value in ILK^+^ cultures incubated without NAC and H_2_O_2_, which is set to 1. Data are means ± SEM (*n* = 3). Statistical significance was calculated using ANOVA.

Excessive generation of reactive oxygen species by oxidants or as a result of UV irradiation induces phosphorylation and activation of JNK and ERK in primary epidermal cells [25]. Therefore, we next investigated if cellular IOS levels are altered in ILK^KO^ keratinocytes by measuring DCFDA fluorescence in intact cells. We observed a 40% increase in fluorescence values in ILK^KO^ cells cultured in normal growth medium, relative to ILK-expressing keratinocytes (Figure 4A). Cellular IOS-associated DCFDA fluorescence remained 40%-60% higher in ILK^KO^ cells throughout a 45-min time course of measurements (Figure 4A). Incubation of the cells with H_2_O_2_ resulted in time- and concentration-dependent increases in DCFDA-associated fluorescence in both cell types (Figure 4A, 4B). However, DCFDA-associated fluorescence values in ILK^KO^ keratinocytes were about 1.5-fold higher than in ILK^+^ cells at all H_2_O_2_ concentrations tested. We also observed that DCFDA fluorescence in ILK^KO^ cells treated with the glutathione precursor NAC decreased to levels indistinguishable from those in ILK-expressing keratinocytes (Figure 4C). However, upon a 5- or 45-min H_2_O_2_ challenge, NAC treatment was not sufficient to decrease IOS levels in ILK^KO^ cells to the same extent observed in ILK^+^ cells (Figure 4C and data not shown). Although the changes in DCFDA fluorescence observed in the presence of H_2_O_2_ likely reflect IOS production from cellular processes, as well as direct formation of hydroxyl radicals from chemical breakdown of the exogenously added hydrogen peroxide, collectively, our data are consistent with the notion that the absence of ILK leads to accumulation of IOS and an altered cellular redox state in keratinocytes.

**Figure 5:**
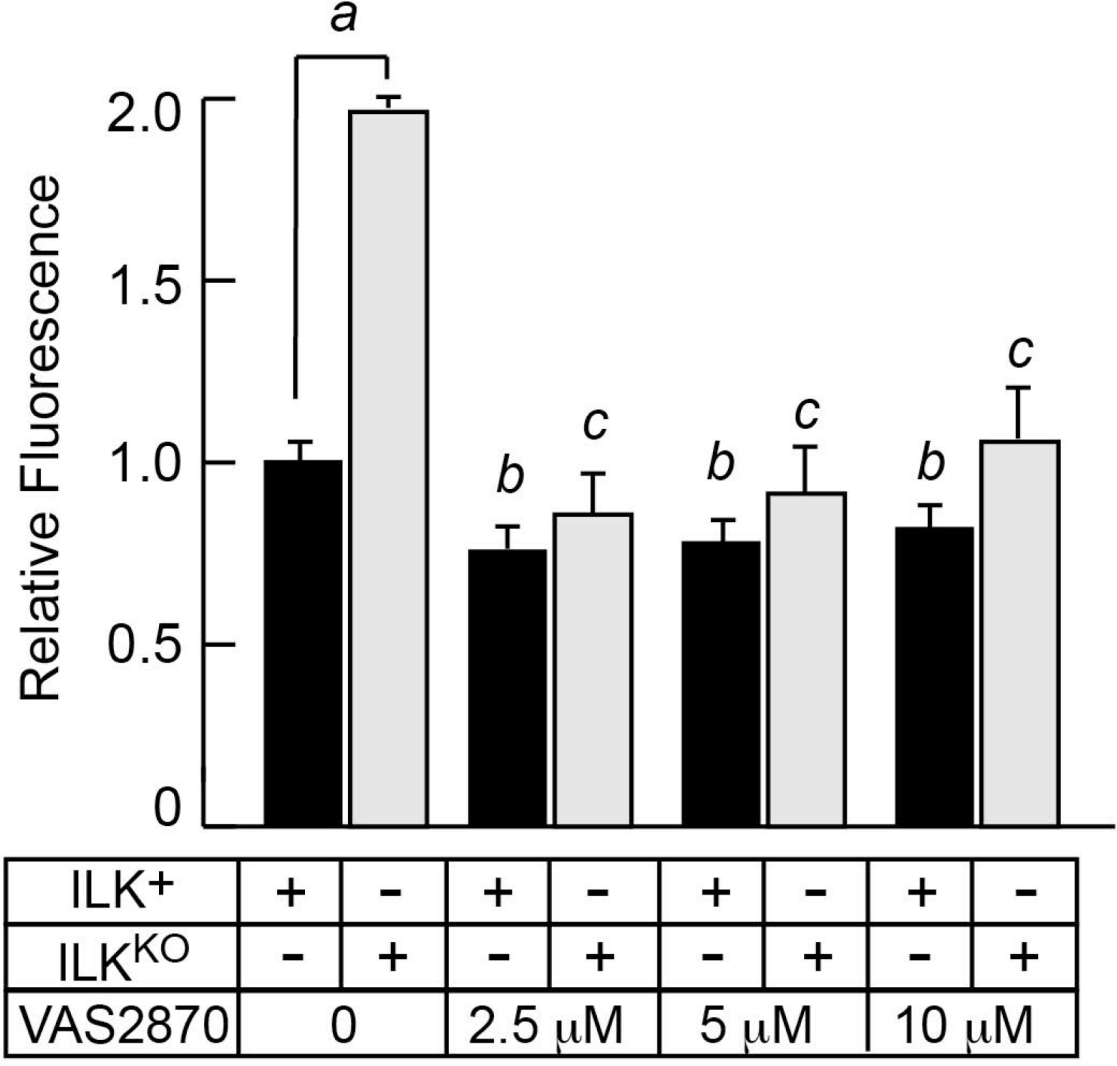
Inhibition of NADPH oxidase activity decreases intracellular oxidant species in ILK^KO^ keratinocytes Keratinocytes were cultured in the presence of the indicated concentrations of VAS2870 for 24 h. After removal of the drug, the cells were loaded by incubation at 37° C in the presence of 10 μM DCFDA. Following DCFDA removal, fluorescence values were determined after 45 min, and were normalized for cell number. The results are expressed as fold-change relative to the normalized fluorescence value in ILK^+^ cultures incubated without VAS2870, which is set to 1. Data are means + SEM (*n* = 3). ^a^*P* < 0.05, ^b^*P* < 0.05 relative to ILK^+^ cells in the absence of VAS2870, ^c^*P* < 0.05 relative to ILK^KO^ cells in the absence of VAS2870 (ANOVA).

The NADPH oxidase family of transmembrane enzymes, can function as a primary source of oxidant species generation under normal and pathological conditions [28]. This enzyme family is composed of six subunits, which can associate to form an active complex that generates O_2_^-^ from oxygen and NADPH [28]. There are seven homologues of the catalytic subunit, termed NOX-1 through -5, Duox-1 and Duox-2. Epidermal keratinocytes are known to express NOX-1, NOX-2 and NOX-4, and abnormal NOX-1 activation occurs in keratinocytes in which DNA repair pathways are impaired [29]. To investigate if NOX activity contributed to higher IOS levels in the absence of ILK, we treated keratinocytes with the pan-NOX inhibitor VAS2870. Treatment of normal keratinocytes with VAS2870 decreased DCFDA fluorescence by about 25%. Significantly, the presence of this inhibitor in ILK^KO^ cultures decreased DCFDA fluorescence about 60%, and to levels indistinguishable to those found in normal keratinocytes (Figure 5). Thus, NADPH oxidase activity likely contributes to the excess IOS observed as a result of *Ilk* gene inactivation.

### Increased damage and impaired repair of genomic DNA in the absence of ILK

**Figure 6:**
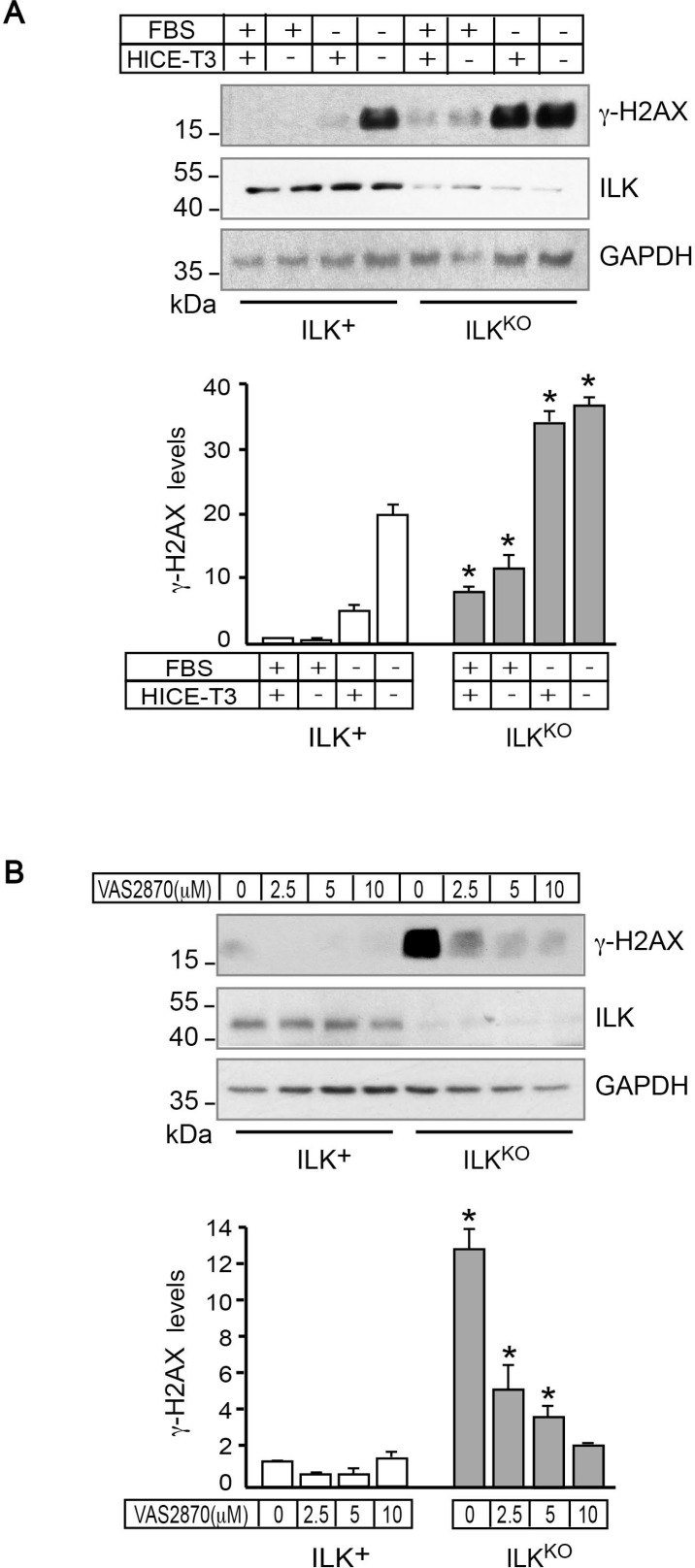
NADPH oxidase-dependent presence of γH2AX in ILK^KO^ keratinocytes (**A**) ILK^+^ and ILK^KO^ keratinocytes were cultured with the indicated growth additives for 24 h. Cell lysates were prepared and 10 µg/sample were analyzed by immunoblot using antibodies against the indicated proteins; GAPDH served as loading control. The histograms under the representative immunoblot show γH2AX densitometric quantification (mean ± SD, *n* = 3), and are expressed relative to levels in ILK-expressing cells cultured in growth medium containing FBS and HICE-T3, set to 1. ^*^P < 0.05 (ANOVA), relative to γH2AX levels in ILK^+^ cells cultured in the corresponding culture condition. (**B**) Keratinocytes were cultured in the presence of the indicated concentrations of VAS2870 for 24 h, and the indicated proteins were analyzed as described in (A), using 80 µg of protein lysate/sample, to better visualize dose-dependent VAS2870 reductions in γH2AX. The histograms under the representative immunoblot show γH2AX densitometric quantification (mean ± SD, *n* = 3), and are expressed relative to the level in ILK^+^ cells cultured in the absence of drug, which is set to 1. ^*^P < 0.05 (ANOVA).

Abnormal IOS generation can promote the oxidation of nuclear DNA in keratinocytes and other cell types [30]. DNA lesions produced by reactive oxygen species and other oxidants can include single- and double-strand breaks, and are associated with the formation of phosphorylated histone H2AX (γH2AX) during the DNA damage response [31, 32]. Examination of these cells by immunofluorescence microscopy revealed that 0.2%-0.5% of ILK^+^ cells exhibited γH2AX foci (data not shown). Analysis of lysates prepared from ILK-expressing keratinocytes cultured under optimal growth conditions showed very low levels of γH2AX. Culture of these cells either in serum- or in growth factor-free medium resulted in little, if any, increases in γH2AX, whereas culture in the absence of both serum and growth factors caused a marked increase in γH2AX abundance (Figure 6A). In stark contrast, the levels of γH2AX in ILK^KO^ keratinocytes were consistently 7- to 10-fold higher than in normal cells, irrespective of whether they were cultured in optimal, in serum- or in growth factor-deprivation conditions (Figure 6A). To examine the involvement of increased IOS levels in H2AX phosphorylation, we also measured γH2AX abundance in cells treated with VAS2870 for 24 h. Although NOX inhibition did not significantly alter the low levels of γH2AX in ILK-expressing keratinocytes, it reduced them in ILK^KO^ cells about 7-fold (Figure 6B), indicating a causative link between excessive IOS production via NOX enzymes and activation of DNA damage responses in these cells.

**Figure 7:**
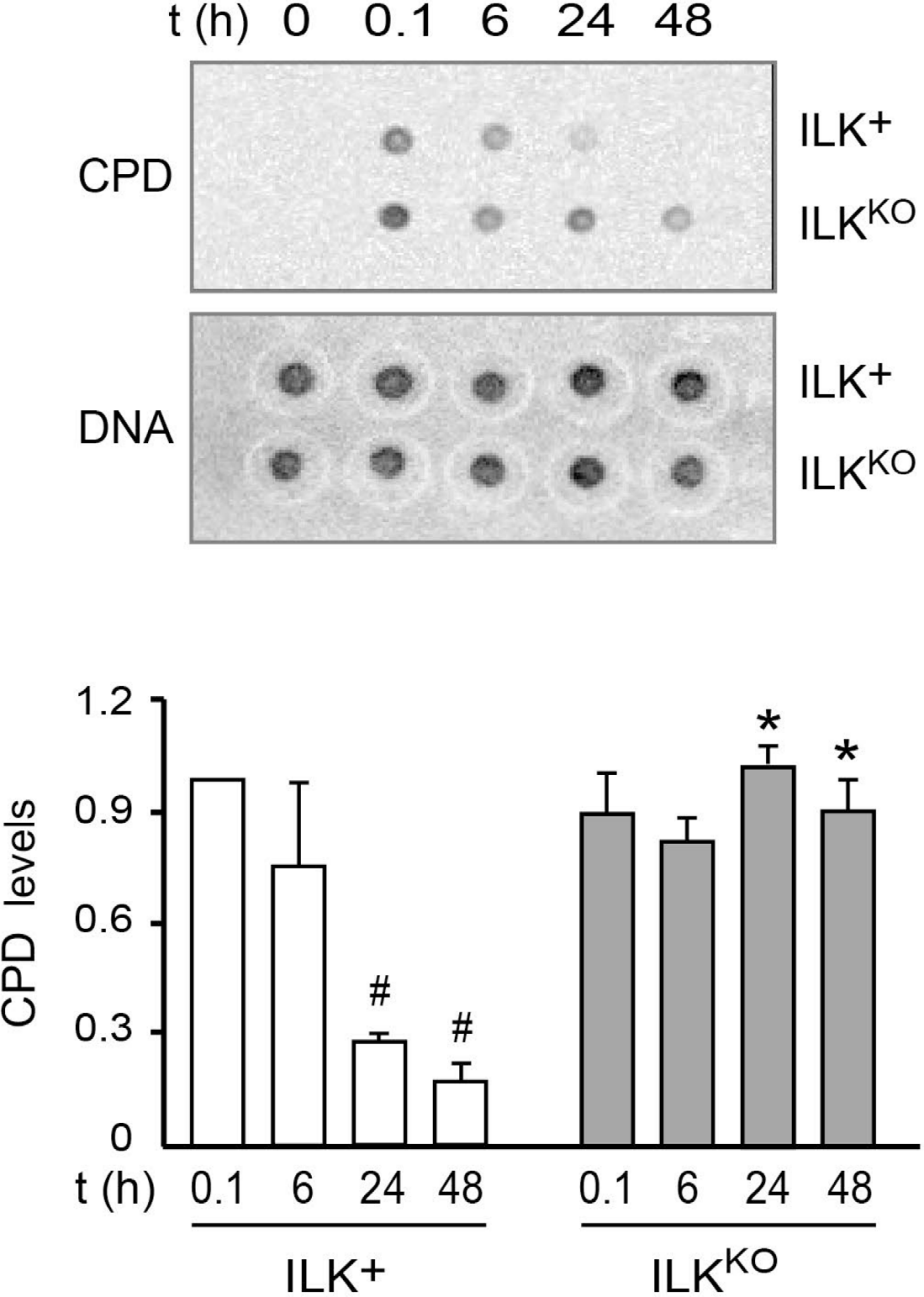
Defective DNA repair following UV-induced damage in ILK^KO^ keratinocytes Keratinocytes were subjected to UVB irradiation (50 J/m^2^). Genomic DNA was isolated at the indicated times thereafter, and analyzed for the presence of CPD as described in “Materials and Methods”. CPD levels were normalized to the amount of double-stranded DNA spotted on a replicate membrane. The histograms under the representative blots show the fraction of normalized CPD signal remaining after 6, 24 and 48 h, relative to CPD levels measured 0.1 h after irradiation (set to 1). The results are expressed as mean ± SD (*n* = 3). ^#^*P* < 0.05 relative to CPD in ILK^+^ cells at *t* = 0.1; ^*^P < 0.05 relative to CPD in ILK^+^ at each corresponding time following UVB irradiation (ANOVA).

Due to their location on the body surface, epidermal keratinocytes are constantly exposed to damage from UV light and environmental oxygen. Given the increased oxidative stress of ILK^KO^ cells, we next examined their capacity to repair UV-induced DNA damage. To this end, we subjected the cells to UV irradiation to induce CPD formation in genomic DNA, and measured their ability to repair those lesions as a function of time. ILK-expressing keratinocytes showed maximum CPD levels immediately after UV irradiation, which decreased by 80% and 95%, respectively, after 24 h and 48 h (Figure 7). UV irradiation of ILK^KO^ cells produced similar initial CPD levels to those observed in normal keratinocytes. However, CPD abundance in these cells remained unchanged even 48 h after irradiation, indicating that lack of ILK expression also reduces the DNA repair capacity of the cells.

## DISCUSSION

This study reveals important and novel mechanistic insights into how oxidative stress in keratinocytes is regulated, with specific emphasis on the protective role of ILK against oxidative stress and in maintaining genomic integrity. Our data lead to two major conclusions. First, ILK is involved in protecting cells from growth factor deprivation and modulating processes that maintain proper redox balance. Second, ILK is required for normal genomic integrity and repair after UVB irradiation.

Loss of ILK during embryogenesis also results in abnormal distribution of and signalling through β1 integrins, which leads to hair follicle developmental arrest [20]. In the interfollicular epidermis, ILK-deficient keratinocytes exhibit defects in attachment to the basement membrane [33]. In culture, ILK-deficient keratinocytes exhibit defects in cell spreading and fail to form normal focal adhesions [11, 33]. Keratinocytes can respond to the loss of extracellular matrix attachment in different manners. *In vivo,* undifferentiated keratinocytes in the lowermost basal layer of the epidermis express high levels of α6β4 and αβ1 integrins, which contribute to cell attachment to the underlying basement membrane [34]. Targeted inactivation of *Ilk* in mouse epidermis yields abnormalities that closely phenocopy those observed upon inactivation of the *Intb1* gene [35, 36], suggesting that ILK is likely a critical transducer of a variety of signals received through β1 integrins in undifferentiated keratinocytes, including those implicated in responses to growth factor restriction. Although the interruption of ILK-mediated survival pathways in keratinocytes cultured under serum and/or growth factor deprivation conditions triggers activation of caspase-dependent apoptosis, this process does not appear to be associated with changes in Akt activation. Stimulation of β1 integrins in some cell types causes Akt Ser473 phosphorylation, which can then participate in the activation of cell survival pathways, although contrasting evidence for the role of ILK in Akt regulation has been extensively reported [37, 38]. Our data show that ILK is dispensable for Akt phosphorylation in undifferentiated keratinocytes. Similarly, in HeLa and MCF-7 epithelial cells, Akt phosphorylation does not depend on ILK, but instead requires RICTOR-mTor complexes [39]. Because inhibition of mTor reduces cell survival in response to UV irradiation in epidermal keratinocytes [40], an important aspect for future research will be to determine whether the reduced viability of ILK-deficient keratinocytes is associated with abnormalities in RICTOR and/or mTor.

Oxidant species are required as signal messengers in many physiological processes. Significantly, interactions between cellular integrins and their surrounding extracellular matrix can also elicit production of IOS [41]. In an apparently paradoxical manner, impaired responses to integrins due to cell detachment can give rise to pathological, elevated levels of IOS production through alterations in several metabolic pathways [41]. For example, a key metabolic alteration in detached mammary epithelial cells is impaired glucose uptake, which triggers a chain of events resulting in reduced levels of ATP and NADPH [22]. Given that the latter serves as a reducing agent and reactive oxygen species scavenger, IOS levels increase under these conditions, although such increases can be prevented by exogenous antioxidants, such as NAC. Similarly, inactivation of *Ilk* in epidermal keratinocytes is accompanied by increases in IOS levels that can be reversed in the presence of NAC, an agent that increases glutathione levels in cultured keratinocytes, affording them protection against UV photodamage [42]. Although glutathione, NADPH levels and glucose uptake in ILK-deficient keratinocytes remain to be measured, we have also observed that lack of ILK in cultured keratinocytes is accompanied by significantly reduced cellular ATP levels (M. Im, S. Sayedyahossein and L. Dagnino, unpublished observations).

A key finding of our study is that inactivation of *Ilk* also leads to increased levels of γH2AX, indicative of DNA lesions and/or activation of the DNA damage response (DDR). The physiological and pathological roles of NOX enzymes in keratinocytes, in which only the isoforms NOX-1, -2 and -4 have been detected [43], are not fully understood. IOS production by NOX-1 in response to growth factors, and possibly α3β1 integrin stimulation too, requires Tiam1-mediated Rac1 activation in keratinocytes [26]. Although ILK is an essential upstream mediator of Rac1 activation in these cells in response to growth factor- and α3β1 integrin-stimulation [11, 15, 33, 44, 45], the seemingly paradoxical elevation in IOS levels in ILK-deficient keratinocytes cultured in the presence of growth factors suggests that either normal Rac1 activation is dispensable in this process, or that it occurs through ILK-independent pathways. Alternatively, initial supranormal IOS production in ILK-deficient keratinocytes, potentially arising from abnormal integrin- and growth factor-mediated signalling, may lead to DNA and proteome damage, thus initiating a self-sustaining cycle that may involve the activation of one or several molecular pathways. It is well established that DNA lesions trigger stimulation of DDR. Activation of DDR effectors can also induce metabolic IOS production, thus constituting an amplification loop between IOS and the DDR itself [46]. In particular, the DDR component γH2AX induces IOS increases through mechanisms that involve sequestration of 14-3-3ζ and consequent activation of Rac1 and NOX-1 [47]. In keratinocytes, the presence of unrepaired damaged DNA also leads to stimulation of DNA-protein kinase, which, in turn, promotes Akt activation. The latter leads to induction of NOX-1, generation of cytoplasmic IOS and enhanced DNA oxidation, which then causes further IOS generation and sustained macromolecular damage [30]. Given that ILK^KO^ cells also exhibit defects in proliferation and increased sensitivity to apoptotic stimuli, it is possible that these defects also contribute to the observed changes in IOS and DNA integrity observed. Although it is difficult to accurately determine the extent of such contributions, our observation that inhibition of NADPH oxidases is accompanied by reduction of IOS would suggest that some of the apoptosis-related events we observed in ILK-deficient keratinocytes might occur downstream from abnormal NOX dependent IOS production.

Double DNA strand breaks are some of the most ominous forms of DNA damage, and the generation of γH2AX serves to recruit repair factors to protect genomic integrity. Of note, γH2AX can also serve as a biomarker of other types of DNA lesions and/or DDR activation [32, 48], and studies to address in detail the type of DNA damage or DDR present in ILK^KO^ cells and the mechanisms involved will be key areas for future research. The constitutively high levels of γH2AX in ILK-deficient keratinocytes suggest that these cells may also be more susceptible to genomic instability upon oxidative insults, with potential outcomes that include apoptosis, senescence and/or carcinogenic transformation. In support of this notion, we demonstrated that the ability of ILK-deficient cells to repair DNA photolesions is reduced. Additionally, given that ILK^KO^ cells are more susceptible to be negatively affected by various stressors, as seen with growth factor deprivation, it is possible that these properties may contribute, at least in part, to their reduced ability to remove CPD lesions following UV irradiation. The direct analyses of the excision-repair components in these cells should address these issues in the future. Recently, NOX-1 expression and activity were found to increase as a result of DNA lesions arising from UVB photodamage or from the genetic impairment of DNA repair efficiency, such as during loss of normal XPC function [7, 29]. In addition, blockade of NOX-1 activation in response to UVB irradiation increases DNA nucleotide excision repair, decreasing genomic instability and formation of cutaneous squamous cell carcinomas [7]. Conversely, impaired DNA repair efficiency causes NOX-1 activation, subsequently leading to increased cytoplasmic IOS production, and altered mitochondrial bioenergetics [29]. Significantly, similar to ILK-deficient keratinocytes, inactivation of the *Rac1* gene in these cells results in attenuated DDR responses and increased susceptibility to carcinogenesis following UV irradiation [49]. Given that ILK is required for Rac1 activation in several processes independent of integrin signalling [45], it will be important to determine whether Rac1 is a downstream ILK effector in DNA repair responses following UV irradiation in epidermal cells.

Under normal conditions, the outermost regions of the skin are supplied by external oxygen, which diffuses from the air to a depth of about 400 µm [50]. The non-vascularized epidermis, with an average thickness of 40–70 µm [51], receives its oxygen supply exclusively through atmospheric oxygen. Although accurate measurements of partial oxygen pressure (pO_2_) in the skin are technically challenging, a pO_2_ of about 120 Torr has been estimated for the skin at a depth of 70 µm, where the lowermost basal epidermal keratinocyte are found [50]. This value is close to the atmospheric pO_2_ at sea level of about 160 Torr. Thus, unlike many other tissues in the body, including the innermost regions of the dermis, epidermal keratinocytes are physiologically exposed to near atmospheric pO_2_ values [50], which closely mirror the conditions of our experiments. Intracellular oxidant species and antioxidant levels determine the redox state in tissues and cells, and the location of epidermal keratinocytes on the body surface results in their constant exposure to IOS-generating external stimuli. Thus, the ability of these cells to regulate their redox state is key for epidermal homeostasis. We have now shown that, in addition to its central roles in epidermal development and physical integrity, ILK is also critical for the maintenance in this tissue of redox equilibrium and proper function of DNA repair pathways in response to UV-induced photodamage.

## MATERIALS AND METHODS

### Mouse strains

The generation and genotyping of mice with epidermis-restricted inactivation of the *Ilk* gene has been described [20, 33].All animal experiments were approved by the University of Western Ontario Animal Care Committee (Protocol No. 2015-021), in accordance with regulations and guidelines from the Canadian Council on Animal Care.

### Reagents and antibodies

Cholera toxin (100) and insulin (16634) were from List Biological (Campbell, CA, USA) and Invitrogen (Carlsbad, CA, USA), respectively. Chelex 100 resin was purchased from Biorad (Mississauga, ON, Canada). Rat tail collagen I was from BD Biosciences (Bedford, MA), and 2′,7′-dichlorofluorescein diacetate (DCFDA) was from Calbiochem (EMD Millipore; Billerica, MA). VAS2070 (492000) was from EMD Millipore Corporation (Billerica, MA). All other reagents were purchased from Sigma (St. Louis, MO, USA). The primary antibodies used were: Bax (sc-493, Santa Cruz Biotechnology, Santa Cruz, CA), 5’-bromodeoxyuridine (BrdU, G3G4, Developmental Studies Hybridoma Bank, Iowa City, IA), cyclobutane pyrimidine dimers (CPD; Clone TDM2, CAC-NM-DND-001, Cosmo Bio International, Tokyo, Japan), double-strand DNA (dsDNA) (ab27156, Abcam, Cambridge, MA), GAPDH (ADI CSA 335, Enzo Life Sciences, Ann Arbour, MA), ILK (611802, BD Transduction Laboratories, Lexington, KY), γ-tubulin (T6557, Sigma). The following antibodies were purchased from Cell Signaling Technology (Beverly, MA): Cleaved caspase-3 (9661), cleaved PARP-1 (9544), total Akt (9272), phospho-Akt (Thr308, 9275), total p38 (9212), phospho-p38 (Thr180/Tyr182, 4511), γH2AX (9718), total p42/p44 ERK (9101), phospho-p42/p44 ERK (Thr202/Tyr185, 4370), total SAPK/JNK (9252), and phospho-SAPK/JNK (Thr183/Tyr185, 4668). Alexa Fluor-conjugated goat anti-mouse IgG, goat anti-rabbit IgG and phalloidin (A12381) were purchased from Molecular Probes/Invitrogen (Eugene, OR).

### Keratinocyte isolation and culture

Keratinocytes were isolated from 3-day-old *Ilk*^*tm1Star*^ mice (hereafter termed ILK^f/f^), with two functional alleles of the *Ilk* gene [52], from *K14Cre; Ilk*^*f/+*^ mice (ILK-expressing, with one functional *Ilk* allele, hereafter termed ILK^+^) or *K14Cre; Ilk*^*f/f*^ littermates (ILK-deficient, hereafter termed ILK^KO^), as described [44, 53, 54]. Keratinocytes were seeded either onto cell culture plates or on acid-washed glass coverslips coated with poly-L-lysine (1 mg/ml) and rat-tail collagen type I (5 µg/cm^2^). All experiments were conducted with keratinocytes cultured 48–72 h after isolation, at ≤80% confluence. In experiments using H_2_O_2_, the latter was diluted in growth medium immediately before addition to cells. N-acetylcysteine (NAC) stock solutions (125 mM) were prepared in phosphate-buffered saline (PBS), and the pH was adjusted to 7.4 prior to addition to cultures. All experiments were conducted 3–5 times with triplicate samples and independent cell isolates.

### Analysis of cell proliferation and apoptosis

To determine BrdU incorporation into DNA, cells (1 × 10^5^ cells/cm^2^) were incubated for 2 h in the presence of 10 μM BrdU. The cells were fixed in freshly diluted 4% paraformaldehyde (PFA, 40 min, 4° C) and permeabilized in 0.1% Triton-X 100. Nuclear DNA was denatured by incubation in 2 M HCl (20 min, 22° C). The cells were probed with anti-BrdU antibodies, and the fraction of cells exhibiting BrdU immunoreactivity was determined from immunofluorescence micrographs as described [55]. Apoptosis was measured by colorimetric quantification of cytoplasmic mono- and oligonucleosomes, using a Cell Death ELISA kit (Roche Applied Science), as described [36].

### Immunoblot analysis

The preparation of cell lysates and their analysis by immunoblot has been described [16, 56]. For analysis of phosphorylated proteins, cell lysates were prepared using Phosphosafe extraction buffer (EMD Millipore) supplemented with protease inhibitors [57]. Protein levels in immunoblots were determined by densitometric analysis, using Image J (NIH), or from images acquired on a VersaDoc imaging system equipped with Quantity One software for quantification (BioRad Laboratories, Hercules, CA).

### Immunofluorescence microscopy

Following a rinse with PBS, keratinocyte specimens were permeabilized and fixed by incubation with PBS containing 0.1% Triton X-100 and freshly-diluted 4% PFA for 20 min (22° C) [55, 58]. The cells were rinsed twice with PBS and blocked overnight (4° C) with PBS containing 5% nonfat dry milk and 5% goat serum. After three PBS washes, the cells were incubated with primary antibody for 1 h (22° C). After three 20-min PBS washes, the cells were probed with AlexaFluor-conjugated secondary antibody, at 22° C for 1 h. After removal of the secondary antibody, the cells were rinsed twice, incubated with Hoechst 33342 (10 μg/ml) in PBS for 5 min at 22° C, rinsed five times, and mounted in Immu-Mount medium (Thermo Scientific, Pittsburgh, PA). Immunofluorescence micrographs were obtained with a Leica DMIRBE fluorescence microscope (Leica Microsystems, Wetzlar, Germany) equipped with an Orca-ER digital camera (Hamamatsu Photonics, Hamamatsu, Japan), using Volocity 4.3.2 software. At least 100 individual cells of each genotype were scored in each experiment, and the experiments were repeated with 4 independent cell isolates.

### Measurement of intracellular oxidant species

To evaluate IOS production, keratinocytes seeded in 96-well plates (1 × 10^5^ cells/0.32 cm^2^) were cultured in the presence of 10 μM DCFDA at 37° C for 45 min, protected from light. Following a PBS wash and addition of PBS containing 8% FBS, fluorescence was measured at timed intervals thereafter using a microplate reader (490 nm excitation and 535 nm emission) and Softmax Pro V. 5 software. Replicate samples were used to determine the number of cells in the samples, and fluorescence values were normalized to cell number. As indicated in some experiments, cells were incubated in the presence of 2 mM or 5 mM NAC for 45 min at 37° C prior to DCFDA addition. To measure IOS generation in response to hydrogen peroxide, DCFDA-treated keratinocytes were incubated in the presence of H_2_O_2_, and DFCDA-associated fluorescence was measured at timed intervals thereafter.

### UV irradiation and CPD measurements

The levels of DNA-associated CPD were determined as previously described [59]. Keratinocytes plated in 6-cm culture dishes at a density of 3–3.5 × 10^6^ cells/dish were rinsed with PBS and irradiated with 50 mJ/m^2^ UVB light using a UVM-28 EL series lamp (302 nm, UVP, Upland, CA). The cells were cultured and genomic DNA was isolated at timed intervals following UVB irradiation, using a DNeasy Blood and Tissue Kit (69506, Qiagen, Germantown, MD). DNA (200 ng) was then diluted in TE buffer (10 mM Tris-HCl, 1 mM EDTA, pH 7.5), denatured by heating to 100° C and spotted onto Hybond-N+ membranes (Amersham/GE Health Care Life Sciences, Mississauga, Canada). The membranes were baked (80° C, 1 h). The DNA was denatured by incubation in 0.4 M NaOH (20 min, 22° C). The membranes were rinsed with PBS containing 0.2% Tween-20, and probed with anti-CPD antibodies, as described for the immunoblot analyses. DNA loading was normalized probing replicate membranes spotted with non-denatured DNA, using antibodies against double-strand DNA.

### Statistical analysis

Statistical analyses were conducted on experiments of 3–5 biological replicates obtained from independent cell isolates, and analyzed by one-way analysis of variance (ANOVA) with Bonferroni post-hoc test, or two-tailed paired *t*-test, as appropriate, using GraphPad Prism version 5. Significance was set at *P* < 0.05.

## Abbreviations

ANOVA: analysis of variance
BrdU: 5-bromo-2’-deoxyuridine
CPD: cyclobutane pyrimidine dimers
DCFDA: 2’,7’-dichlorofluorescein diacetate
DDR: DNA damage response
dsDNA: double-strand DNA
FBS: fetal bovine serum
GAPDH: glyceraldehyde 3-phosphodehydrogenase
ILK: integrin-linked kinase
IOS: intracellular oxidant species
MAPK: mitogen-activated protein kinase
PBS: phosphate-buffered saline
